# Detecting drug communities and predicting comprehensive drug–drug interactions via balance regularized semi-nonnegative matrix factorization

**DOI:** 10.1186/s13321-019-0352-9

**Published:** 2019-04-08

**Authors:** Jian-Yu Shi, Kui-Tao Mao, Hui Yu, Siu-Ming Yiu

**Affiliations:** 1 School of Life Sciences, Northwestern Polytechnical University, Xi’an, China; 2 School of Computer Science, Northwestern Polytechnical University, Xi’an, China; 3 Department of Computer Science, The University of Hong Kong, Hong Kong, China

**Keywords:** Drug–drug interaction, Weak balance theory, Semi-nonnegative matrix factorization, Regularization, Community

## Abstract

**Background:**

Because drug–drug interactions (DDIs) may cause adverse drug reactions or contribute to complex-disease treatments, it is important to identify DDIs before multiple-drug medications are prescribed. As the alternative of high-cost experimental identifications, computational approaches provide a much cheaper screening for potential DDIs on a large scale manner. Nevertheless, most of them only predict whether or not one drug interacts with another, but neglect their enhancive (positive) and depressive (negative) changes of pharmacological effects. Moreover, these comprehensive DDIs do not occur at random, but exhibit a weakly balanced relationship (a structural property when considering the DDI network), which would help understand how high-order DDIs work.

**Results:**

This work exploits the intrinsically structural relationship to solve two tasks, including drug community detection as well as comprehensive DDI prediction in the cold-start scenario. Accordingly, we first design a balance regularized semi-nonnegative matrix factorization (BRSNMF) to partition the drugs into communities. Then, to predict enhancive and degressive DDIs in the cold-start scenario, we develop a BRSNMF-based predictive approach, which technically leverages drug-binding proteins (DBP) as features to associate new drugs (having no known DDI) with other drugs (having known DDIs). Our experiments demonstrate that BRSNMF can generate the drug communities, which exhibit more reasonable sizes, the property of weak balance as well as pharmacological significances. Moreover, they demonstrate the superiority of DBP features and the inspiring ability of the BRSNMF-based predictive approach on comprehensive DDI prediction with 94% accuracy among top-50 predicted enhancive and 86% accuracy among bottom-50 predicted degressive DDIs.

**Conclusions:**

Owing to the regularization of the weak balance property of the comprehensive DDI network into semi-nonnegative matrix factorization, our proposed BRSNMF is able to not only generate better drug communities but also provide an inspiring comprehensive DDI prediction in the cold-start scenario.

**Electronic supplementary material:**

The online version of this article (10.1186/s13321-019-0352-9) contains supplementary material, which is available to authorized users.

## Introduction

When two or more drugs are taken together, their pharmacological effects or behaviors would be unexpectedly influenced by each other [1]. Such an influence is termed as Drug–Drug Interaction (DDI), which would reduce drug efficacy, increase unexpected toxicities, or induce other adverse drug reactions among the co-prescribed drugs. Unidentified DDIs occur frequently in clinical usages. There exist ~ 15 DDIs out of every 100 drug pairs on average among approved small molecular drugs in DrugBank [2]. They would put patients, who are treated with multiple-drug medications, in an unsafe situation [3–6]. Moreover, understanding DDI is the first step towards drug combination, which involves usually high-order DDIs [7] and becomes one of the promising treatments for multifactorial complex diseases [8]. Consequently, there is an urgent need to analyze and identify DDIs before clinical co-medications are administered. However, traditional experimental approaches for DDI identification (e.g. testing cytochrome P450 [9] or transporter-associated interactions [10]) have high cost and long duration [11]. So far, only a few DDIs could be identified during drug development (usually the clinical trial phase), some of them are reported after the drugs are approved, and many are found in post-market surveillance.

Computational approaches provide a promising alternative to discover potential DDIs on a large scale for further screening and have gained a lot of attention from both academy and industry recently [12, 13]. Data-mining based approaches have been developed for detecting DDIs from different sources [11], such as scientific literatures [14, 15], electronic medical records [16], and the Adverse Event Reporting System of FDA (http://www.fda.gov). Even though these approaches can collect and report known DDIs, they cannot an early warning of potential DDIs before clinical medications are administered. In contrast, machine learning-based approaches (e.g. naïve similarity-based approach [17], network recommendation-based [11], classification-based [18, 19] are able to provide such alerts by utilizing pre-marketed or post-marketed drug attributes [20], such as chemical structures [17, 21], targets [22], hierarchical classification codes [18] and side effects [11, 23].

Most of these existing machine learning-based approaches are designed for conventional binary prediction, which only indicates how likely a pair of drugs is a DDI. But two interacting drugs may change their own pharmacological behaviors or effects (e.g. increasing or decreasing serum concentration) in vivo [21, 23]. For example, the serum concentration of *Quinine* (DrugBank Id: DB00468) increases when it is taken with *Aprepitant* (DB00673), whereas its serum concentration decreases when taken with *Mitotane* (DrugBank Id: DB00648). We refer these two cases of DDIs as an enhancive DDI and a degressive DDI respectively and both of them as comprehensive DDIs, which contains drug changes in terms of pharmacological effects. It is much better to know whether a DDI is enhancive or degressive, especially when making optimal patient care, establishing drug dosage, or finding drug resistance to therapy [24].

**Fig. 1:**
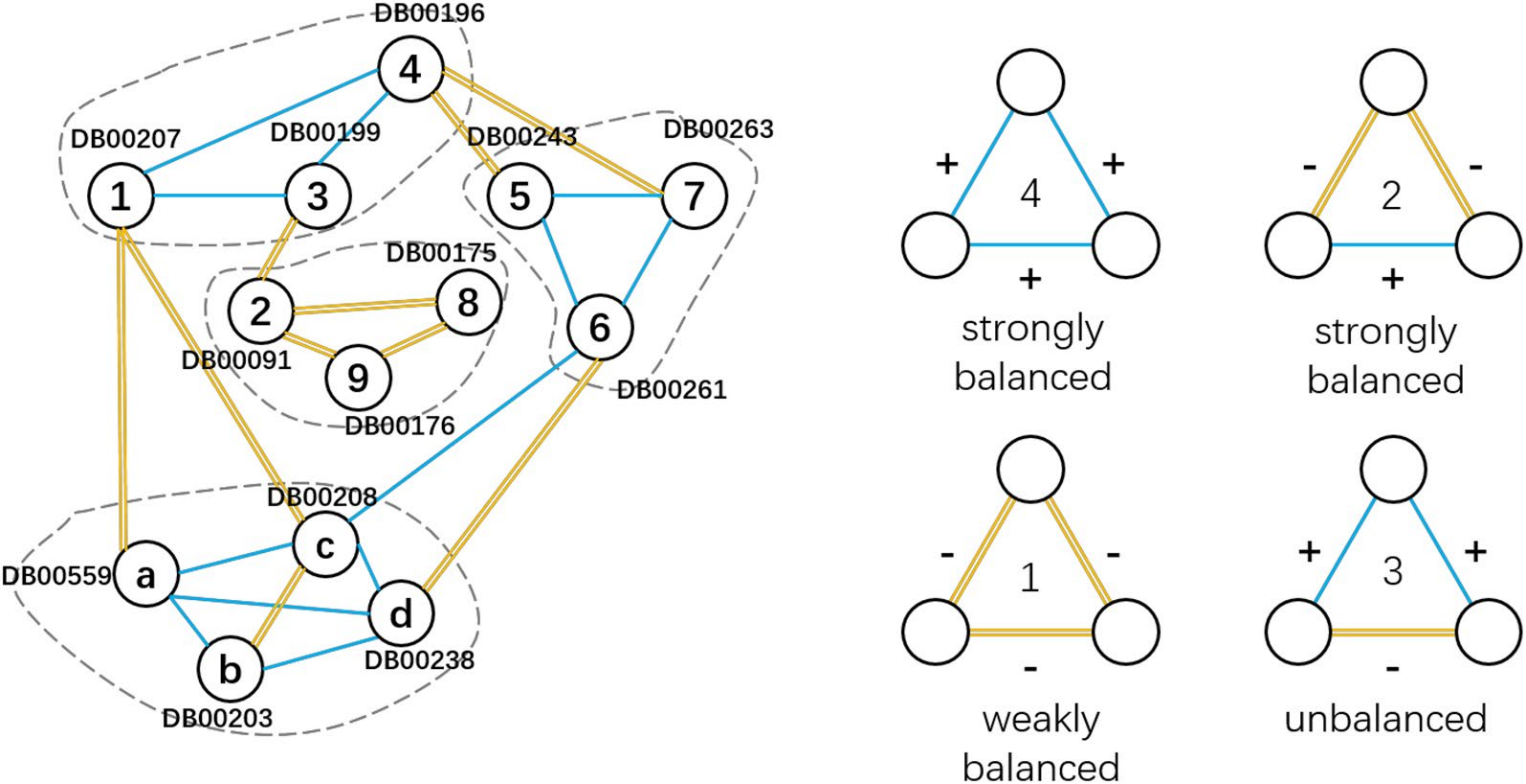
The illustration of a comprehensive DDI network. (1) The left panel shows the DDI network. Four communities are highlighted by dashed curves. Most edges within communities are positive or negative while most edges between communities are negative. Blue single lines and yellow double lines denote enhancive and degressive interactions respectively. (2) The right panel lists four types of triads, including two strongly balanced triads (PPP and NNP), one weakly balanced triad (NNN) and one unbalanced triad (PPN). The numbers in the triads show the number of occurrences of this structure in the network (e.g. PPP appears 4 times in the network)

On the other hand, the occurrence of both enhancive DDIs and degressive DDIs is not random, but exhibits a structural relationship among the drugs when considering the corresponding DDI network [21, 23]. Existing approaches have not yet exploited this structural property, which is, however, one of the most important steps to understand high-order drug interactions treating complex diseases [7]. Two of our recent works [21, 23] attempted to investigate these two issues: (1) predicting comprehensive DDIs instead of binary prediction; and (2) investigating the structural relationship of drugs in a DDI network. One of them proposed a model to predict enhancive and degressive DDIs for different predicting scenarios of new drugs (those with no known DDI) [23]. Another one observed that the numbers of enhancive and degressive DDIs of drugs, as well as their sum/difference, are correlated with drug communities [21]. More importantly, this latter work also reveals that the number of balanced triads (to be defined and explained in Fig. 1) is significantly larger than the number of unbalanced triads in a comprehensive DDI network. This observation is similar to that in signed social networks, which popularly exhibit the nature of global structural balance [25]. Upon the fundamental theorems of Strong Balance [26] and Weak Balance [27], this nature can be leveraged to predict signed links in the social networks [28].

Inspired by signed social networks, this current work exploits the weakly balanced relationship among drugs to solve two tasks: drug community detection as well as comprehensive DDI prediction in the cold-start scenario. The paper is organized as follows. "Methods" section first formulates the community partition in a comprehensive DDI network based on the weak balance theory [27] for signed networks. Then, for the first task, it represents a novel clustering algorithm, balance regularized semi-nonnegative matrix factorization (BRSNMF), which integrate a low-rank matrix decomposition with a weak balance regularization. After that, it depicts a BRSNMF-based predictive approach for the cold-start scenario that requires us to predict potential comprehensive DDIs for newly coming drugs having no known DDI. In Section Results and Discussions, after introducing the weakly balanced phenomenon in a real DDI network, we investigate the advantages of BRSNMF by two comparative experiments. In the first experiment, we compare BRSNMF to the traditional semi-nonnegative matrix factorization by investigating drug numbers, balances, and pharmacological significances across drug communities. In the second one, we compared our features based on drug-binding proteins (DBP) to the popular features based on drug chemical structures (e.g. PubChem fingerprints) under cross-validation. Furthermore, leveraging our DBP features under a version-independent test, we compared our BRSNMF-based approach with the state-of-the-art approach DDINMF [21], which considers nothing about the weakly balanced relationship among drugs. In the last section, we draw our conclusions with discussions.

## Methods

### Community partition in comprehensive DDI network

Without loss of generality, let ${\mathbf{D}} = \left\{ {d_{i} } \right\},i = 1,2, \ldots ,m$ be a set of *m* approved drugs. Their interactions can be accordingly represented as an $m \times m$ symmetric interaction matrix ${\mathbf{A}}_{m \times m} = \left\{ {a_{ij} } \right\}$. For the conventional DDIs, $a_{ij} = 1$ if $d_{i}$ interacts with $d_{j}$, and $a_{ij} = 0$ otherwise. For the comprehensive DDIs, $a_{ij} \in \{ - 1,0, + 1\}$. Again, if $d_{i}$ and $d_{j}$ do not interact with each other, $a_{ij} = 0$. When there is an enhancive DDI or a degressive DDI between $d_{i}$ and $d_{j}$, $a_{ij} = + 1$ or $a_{ij} = - 1$ respectively. The conventional binary DDI matrix ${\mathbf{A}}_{b}$ can be obtained from the comprehensive DDI matrix by setting ${\mathbf{A}}_{b} = Binary({\mathbf{A}})$ (taking the absolute values of all elements). The comprehensive DDI matrix characterizes a signed network $G(N,E)$, in which drugs are nodes and their interactions are edges.

According to Weak Balance Theory [27], the nodes of a weakly balanced signed network can be ideally clustered into *k* groups, such that the edges within groups are positive (enhancive) and the edges between groups are negative (degressive). In such a weakly balanced network, all its *l*-cycles are strongly or weakly balanced. Here, an *l*-cycle is defined as a simple path from some node to itself with length equal to *l*. We mainly consider the case of *l* = 3, where a 3-cycle is called as a triad. There are four kinds of triads, labelled as PPP, NNP, NNN, and PPN respectively, where P denotes positive and N denotes negative edges in a triad (Fig. 1). The first two triads are strongly balanced, the third is weakly balanced and the last is unbalanced. The real-world signed networks (e.g. Epinions and Slashdot) are not purely balanced because they contain some (although much fewer) unbalanced triads (Fig. 1), which are caused by negative edges within groups or positive edges between groups.

Our DDI network is also such a network, which contains significantly more balanced triads than unbalanced triads [21]. We verify our observation using the real data in DrugBank (see "Dataset" section). In our DDI network, we also observe that it may contain a community, in which most edges are negative (i.e. most triads in the community are weakly balanced). Considering the above observations, we generalize the weak balance theory as follows: the nodes of a weakly balanced network can be ideally clustered into k groups, such that most edges within groups are positive (strongly balanced groups) or negative (weakly balanced groups) while most edges between groups are negative. In the context of such a comprehensive DDI network, a drug community is referred to as a cluster, in which the number of balanced *l*-cycles is significantly greater than that of unbalanced *l*-cycles. A real example of a small DDI sub-network illustrates this idea (Fig. 1).

When given a DDI network, our problem can be formulated as a k-way clustering problem (i.e. finding k communities $\{ C_{1} , \ldots ,C_{k} \}$). We anticipate (1) the clustering partitions the network into k evenly distributed drug clusters, of which each contains a sufficient number of drug nodes; (2) more importantly, most interactions within clusters are enhancive or degressive while most interactions between clusters are degressive. This clustering problem is NP-hard [29]. To solve it, we present an approximate solution by designing a low-rank matrix decomposition, which maps the network into a low-dimensional space so as to reveal its underlying weakly balanced structure.

### Clustering by balance regularized semi-nonnegative matrix factorization

For a non-negative matrix A, nonnegative matrix factorization (NMF) decomposes it into two low-rank nonnegative factor matrices W and H, such that ${\mathbf{A}} \approx {\mathbf{WH}}^{T}$. The non-negativity of NMF makes both W and H easier to interpret and provides an inherent clustering, in which the columns of W play the cluster centroids and the rows of H can be viewed as the cluster indicators for the columns of A. Since the strong constraint of non-negativity of A, NMF cannot be applied in many problems (e.g. our problem). To accommodate more scenarios, one of its extensions, semi-nonnegative matrix factorization (Semi-NMF) is proposed for a real matrix A with only one constraint of non-negativity of H [30]. Motivated by Semi-NMF, we design a variant of semi-NMF, which not only inherits the advantages of Semi-NMF but also represents the underlying weakly balanced structure of comprehensive DDI network. The novel Semi-NMF on DDI networks is stated formally as a *k*-way clustering problem in the following.

Given a comprehensive DDI matrix ${\mathbf{A}}_{m \times m} \in {\mathbb{R}}$, we aim to find a community centroid matrix ${\mathbf{W}}_{m \times k} = [{\mathbf{w}}_{1} ,{\mathbf{w}}_{2} , \ldots ,{\mathbf{w}}_{k} ] \in {\mathbb{R}}$ and a community indicator matrix ${\mathbf{H}}_{m \times k} = \{ h_{ij} \} \in {\mathbb{R}}^{ + }$, whose product can well approximate the original matrix ${\mathbf{A}}^{ \pm } \approx {\mathbf{W}}^{ \pm } ({\mathbf{H}}^{ + } )^{T}$, where $k \ll rank({\mathbf{A}})$ and the element $h_{ij}$ denotes the likelihood that node *i* belongs to the *j*th community.

Furthermore, we anticipate that most interactions within drug communities are enhancive and most edges between drug communities are degressive. To avoid partitioning where most clusters contain only a few nodes, we also prefer that each cluster contains substantial nodes. As a result, we introduce two graph regularization items, including a within-community criterion $Gr_{1}$ and a between-community criterion $Gr_{2}$, to encode the balanced structure of DDI network. They are defined as follows:

1 $$Gr_{1} = \hbox{min} \sum\limits_{c = 1}^{k} {\frac{{{\mathbf{h}}_{.c}^{T} {\mathbf{A}}^{ - } {\mathbf{h}}_{.c}^{{}} }}{{{\mathbf{h}}_{.c}^{T} {\mathbf{h}}_{.c}^{{}} }}} ,\quad Gr_{2} = \hbox{min} \sum\limits_{c = 1}^{k} {\frac{{{\mathbf{h}}_{.c}^{T} {\mathbf{L}}^{ + } {\mathbf{h}}_{.c}^{{}} }}{{{\mathbf{h}}_{.c}^{T} {\mathbf{h}}_{.c}^{{}} }}}$$

where ${\mathbf{h}}_{.c}^{{}}$ is the cth column vector in H, ${\mathbf{L}}^{ + } = {\mathbf{D}}^{ + } - {\mathbf{A}}^{ + }$, ${\mathbf{D}}^{ + }$ is the diagonal degree matrix of ${\mathbf{A}}^{ + }$, and $\forall i,j.\;a_{ij}^{ + } = {{(|a_{ij} | + a_{ij} )} \mathord{\left/ {\vphantom {{(|a_{ij} | + a_{ij} )} 2}} \right. \kern-0pt} 2}$, $a_{ij}^{ - } = {{(|a_{ij} | - a_{ij} )} \mathord{\left/ {\vphantom {{(|a_{ij} | - a_{ij} )} 2}} \right. \kern-0pt} 2}$.

Inspired by [31], we combine them together and obtain

2 $$\begin{aligned} Gr & = \hbox{min} \sum\limits_{c = 1}^{k} {\frac{{{\mathbf{h}}_{.c}^{T} ({\mathbf{A}}^{ - } + {\mathbf{L}}^{ + } ){\mathbf{h}}_{.c}^{{}} }}{{{\mathbf{h}}_{.c}^{T} {\mathbf{h}}_{.c}^{{}} }}} \\ & \equiv \hbox{max}\,tr\left( {{\mathbf{H}}^{T} {\mathbf{W}}^{1/2} ({\mathbf{W}}^{ - 1} {\hat{\mathbf{K}}\mathbf{W}}^{ - 1} ){\mathbf{W}}^{1/2} {\mathbf{H}}} \right). \\ \end{aligned}$$

when ${\mathbf{W}} = {\mathbf{I}}$ and ${\hat{\mathbf{K}}} = \sigma {\mathbf{I}} - \eta ({\mathbf{A}}^{ - } + {\mathbf{L}}^{ + } )$, it becomes

3 $$Gr \equiv \hbox{max}\,tr\left( {{\mathbf{H}}^{T} (\sigma {\mathbf{I}} - \eta ({\mathbf{A}}^{ - } + {\mathbf{L}}^{ + } )){\mathbf{H}}} \right) = \hbox{max}\,tr({\mathbf{G}}),$$

where $\sigma ,\eta > 0$ control the sizes of clusters [32].

In addition, we introduce another regularization item $Sr$ to control the sparsity of **H** such that the drug nodes in DDI network belong to as few communities as possible. It is defined as,

4 $$Sr = \sum\limits_{j}^{m} {||{\mathbf{h}}_{j.} ||{}_{1}^{2} } = tr\left( {{\mathbf{H1H}}^{T} } \right) = tr({\mathbf{S}}),$$

where ${\mathbf{1}}$ is the $k \times k$ matrix, of which all elements are 1.

Integrating all the regularization items into the low-rank matrix decomposition, we design the balance regularized semi-nonnegative matrix factorization (BRSNMF) as,

5 $$\begin{aligned} \hbox{min} & \left\| {{\mathbf{A}} - {\mathbf{WH}}^{T} } \right\|_{F}^{2} + \alpha \cdot tr({\mathbf{S}}) - \beta \cdot tr({\mathbf{G}}). \\ s.t. & \quad h_{ij} \ge 0,\quad \forall i,j \in [1, \ldots ,m] \\ \end{aligned}$$

Since the constraint is ${\mathbf{H}} \in {\mathbb{R}}^{ + }$, we leverage the Lagrangian function and the Karush–Kuhn–Tucker conditions to solve it by the updating rules as follows

6 $${\mathbf{W}} \leftarrow {\mathbf{AH}}({\mathbf{H}}^{T} {\mathbf{H}})^{ - 1} ,$$

7 $${\mathbf{H}} \leftarrow {\mathbf{H}} \odot \left( {{\mathbf{N}} \div {\mathbf{D}}} \right)^{1/2}$$

8 $${\mathbf{N}} = ({\mathbf{A}}^{T} {\mathbf{W}})_{ + } + ({\mathbf{HW}}^{T} {\mathbf{W}})_{ - } + \beta \eta ({\mathbf{L}}^{ + } {\mathbf{H}})_{ - } + \beta \sigma {\mathbf{H}},$$

9 $${\mathbf{D}} = ({\mathbf{A}}^{T} {\mathbf{W}})_{ - } + ({\mathbf{HW}}^{T} {\mathbf{W}})_{ + } + \alpha {\mathbf{H1}} + \beta \eta {\mathbf{A}}^{ - } {\mathbf{H}} + \beta \eta ({\mathbf{L}}^{ + } {\mathbf{H}})_{ + }$$

where the operators ${\mathbf{X}}_{ + }^{{}} = {{(|{\mathbf{A}}| + {\mathbf{A}})} \mathord{\left/ {\vphantom {{(|{\mathbf{A}}| + {\mathbf{A}})} 2}} \right. \kern-0pt} 2},{\mathbf{X}}_{ - }^{{}} = {{(|{\mathbf{A}}| - {\mathbf{A}})} \mathord{\left/ {\vphantom {{(|{\mathbf{A}}| - {\mathbf{A}})} 2}} \right. \kern-0pt} 2}$, $|{\mathbf{A}}|$ is the element-wise absolute operation on A, $\odot$ and $\div$ are the element-wise product and division between two matrices. The solution of BRSNMF is presented in Algorithm 1. Obviously, the variant of BRSNMF without *Sr* and *Gr* degrades exactly to Semi-NMF. More technical details about Semi-NMF can be found in [21, 33]. Similar to NMF and Semi-NMF, BRSNMF provides an intrinsic clustering, where the columns of **W** play as cluster centroids and the rows of **H** can be viewed as cluster indicators.

To reflect how well a signed network is partitioned into communities, the clustering is globally measured by a community balance index *CBI*, which is a community size-weighted average number of balanced triads in community. It is defined as

10 $$CBI = \frac{{\sum\nolimits_{c = 1}^{k} {n_{c} *\left( {1 - \frac{{\# PPN_{c} }}{{\# triads_{c} }}} \right)} }}{{\sum\nolimits_{c = 1}^{k} {n_{c} } }} \times 100\% \in [0,1],$$

where $\# PPN_{c}$ is the number of unbalanced triads and $\# triads_{c}$ is the total number of triads in community *c*, $n_{c}$ denotes the community size and *k* is the total number of communities in the clustering. The greater the value of CBI, the better the clustering.

In addition, we define two local metrics, Community-Within Difference (Δ_*w*_) and Community-Between Difference (Δ_*b*_), as $\Delta_{w} = \ln (R_{e}^{w} ) - \ln (R_{d}^{w} )$ and $\Delta_{b} = \ln (R_{e}^{b} ) - \ln (R_{d}^{b} )$, where $R_{e}^{w}$ is the ratio of enhancive DDIs to all the drug pairs,$R_{d}^{w}$ is the ratio of degressive DDIs to all the drug pairs within a community. Similarly, $R_{e}^{b}$ and $R_{d}^{b}$ are two corresponding ratios between two communities. The larger difference, the more enhancive DDIs; the smaller the difference, the more degressive DDIs.
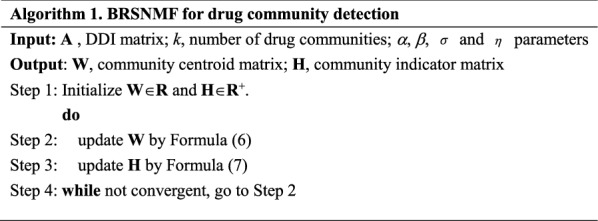


### BRSNMF-based approaches for predicting potential comprehensive DDIs of new drugs

**Fig. 2:**
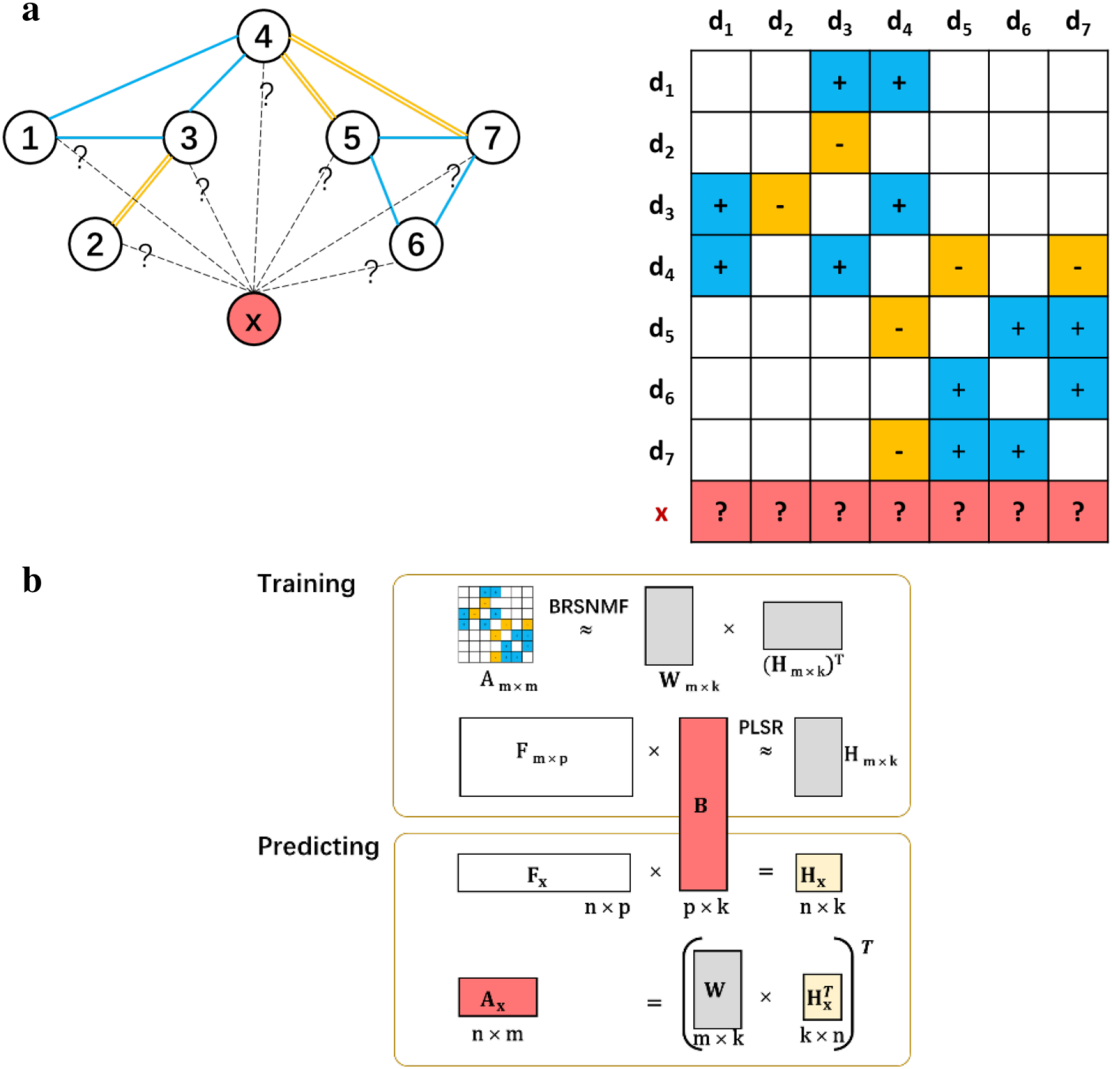
Predicting comprehensive interactions in the cold-start scenario. **a** A toy example of predicting how likely the new drug ‘X’ interacts with other drugs in terms of pharmacological changes. In the network shown in the left panel, the nodes numbered from 1 to 7 are drugs having known comprehensive interactions and the isolated node labeled by ‘X’ denotes the newly given drug having no interactions with the previous drugs. Blue single lines and yellow double lines denote enhancive (positive) and degressive (negative) interactions respectively. The adjacent matrix of the DDI network is shown the right panel. **b** The framework of the BRSNMF-based predictive approach. All the dimensions of the matrices are also listed

In this section, we show how to make use of BRSNMF to predict potential comprehensive DDIs focusing on the scenario of DDI prediction between ‘new drugs’ (without known DDIs) and ‘approved drugs’ (drugs with known DDIs) as the prediction problem is known to be difficult if new drugs are involved (Fig. 2a). New drugs can be regarded as isolated nodes in the DDI network [21]. This prediction scenario is analogous to the well-known cold-start problem in social recommendation [34]. Such a prediction requires additional properties (or features) to relate new drugs with approved drugs. Unlike most of protein–protein interactions or drug–target interactions [35], pharmacological DDIs are not physical interactions (usually related to their chemical structures) between drugs, but indirect interactions which are mediated by proteins. Thus, we use drug-protein binding information as the features of the drugs to relate new drugs with approved drugs in the cold-start scenario. In addition, such a kind of features can capture particular pharmacological meanings of drug communities detected by BRSNMF (see the next section for more details).

We formally state the cold-start prediction problem as follows. Let ${\mathbf{D}} = \left\{ {d_{i} } \right\},i = 1,2, \ldots ,m$ be a set of *m* approved drugs, the interaction matrix of their DDI network be ${\mathbf{A}}_{m \times m} = \left\{ {a_{ij} } \right\}$, and ${\mathbf{D}}_{x} = \left\{ {d_{x} } \right\},x = 1,2, \ldots ,n$ be a set of *n* new drugs. Any of approved drugs ${\mathbf{D}}$ or new drugs ${\mathbf{D}}_{x}$, is represented as a *p*-dimensional feature vector ${\mathbf{f}}_{i} = [f_{1} ,f_{2} , \ldots ,f_{p} ]$. All the drugs in ${\mathbf{D}}$ are sequentially stacked as an $m \times p$ feature matrix ${\mathbf{F}}$. Similarly, the drugs in ${\mathbf{D}}_{x}$ are stacked as an $n \times p$ feature matrix ${\mathbf{F}}_{x}$. Adopting the framework for the cold-start prediction in [21], Our BRSNMF-based approach in the scenario of predicting DDIs for new drugs includes a training phase and a predicting phase as follows and also illustrated in Fig. 2b.

1. In the training phase, the approach obtains a matrix factorization ${\mathbf{A}}_{m \times m} \approx {\mathbf{W}}_{m \times k} \times ({\mathbf{H}}_{m \times k}^{{}} )^{T}$ BRSNMF and a linear regression ${\mathbf{H}}_{m \times k}^{{}} = {\mathbf{F}}_{m \times p}^{{}} \times {\mathbf{B}}_{p \times k}$ by Partial Least Square Regression (PLSR).
2. In the predicting phase, the learned ${\mathbf{B}}_{p \times k}$ firstly maps ${\mathbf{F}}_{x}^{{}}$ into the $n \times k$ latent space by ${\mathbf{H}}_{x}^{{}} = {\mathbf{F}}_{x}^{{}} \times {\mathbf{B}}$. Then the $n \times m$ predicted interactions between the new drug and the approved drugs by
  11 $${\mathbf{A}}_{x}^{{}} = {\mathbf{H}}_{x}^{{}} {\mathbf{W}}^{T} = ({\mathbf{F}}_{x}^{{}} {\mathbf{B}}){\mathbf{W}}^{T} .$$

Specifically, PLSR combines the properties of PCA and multiple regression by projecting the predicted variables (drug cluster indicator matrix H) and the observable variables (features) to a new space, instead of finding hyperplanes of maximum variance between the response and independent variables. Thus, our BRSNMF-based approach, containing PLSR, implicitly considers the feature reduction, and it has only one parameter *k* to be tuned in the training phase (see also "Comprehensive DDI prediction in the cold-start scenario" section).

As shown in Fig. 2, the cold-start scenario requires the prediction of interactions between newly given drugs having no known DDI and a set of drugs interacting with each other in the form of a DDI network. To mimic such a scenario, we remove a part of drugs with their interactions from the dataset and attempt to predict their interactions by Step 2, while using the remaining drugs and their interactions to by Step 1 in each round of cross-validation (CV). There is a slight difference between two typical CVs, leave-one-out CV (LOOCV) and n-fold CV (n-CV). LOOCV removes only one drug in each round whereas n-CV randomly removes 1/n drugs. Their results have no significant difference when the samples are substantial.

The performance of DDI prediction under CV are illustrated by both the receiver operating characteristic curve (ROC) and the precision–recall curve (PR), and measured by the areas under them, denoted AUROC and AUPR respectively. As suggested by [36], AUPR is more appropriate than AUROC when the number of positive instances is significantly less than that of negative instances. The greater the values of AUROC and AUPR are, the better the prediction is. See their detailed calculation in [21]. In addition, under the consideration that non-interactions could be unknown drug pairs, Mean Percentile Ranking (MPR) is used as an extra performance metric when measuring DDI prediction. The smaller the value, the better the prediction. More technical details about MPR can be found in [37, 38].

## Results and discussions

### Dataset

We collect approved small molecular drugs and their DDIs from DrugBank [2, 39]. After collecting DDIs, we label enhancive DDIs by the keyword ‘increase’ or its synonyms and label degressive DDIs by the keywords ‘decrease’ or its synonyms according to the descriptions of DDI respectively. Two datasets, DB_V4 and DB_V5_Ex, are built according to the version of DrugBank as we need to use known DDIs to validate the accuracy of our prediction. All the drugs and DDIs in DB_V4 are included in DrugBank Version 4 [2], while all the drugs in DB_V5_Ex are newly included in DrugBank Version 5 but not found in DB_V4. The DDIs between the drugs in DB_V5_Ex and the drugs in DB_V4 are also extracted from DrugBank Version 5. The information of these two datasets is summarized in Table 1.

**Table 1:** Details of comprehensive DDI network

DB_V4	Number	DB_V5_Ex	Number
#Drug_V4	1562	#Drug_V5_Ex	39
#E-DDI_V4	125,298	#E-DDI_V4_V5_Ex	1077
#D-DDI_V4	55,278	#D-DDI_V4_V5_Ex	1110

*E-DDI* enhancive DDIs, *D-DDI* degressive DDIs, *DDI_V4* the DDIs among the drugs in DB_V4, *DDI_V4_V5_Ex* the DDIs between the drugs in DB_V4 and the drugs in DB_V5_Ex

For all the drugs, we also collect their drug-binding proteins (DBP), including 1213 drug targets and 429 non-target proteins, which play important roles in pharmacodynamic and pharmacokinetic processes of drugs. These proteins are used to investigate the pharmacological significance and leveraged as features so as to associate new drugs having no known with drugs having known DDIs in the prediction of comprehensive DDI. In the following sections, DB_V4 is first used to detect pharmacological communities ("Drug community partition" section). Then, it is used to validate the effectiveness of DBP features and train a predictive model of comprehensive DDIs while DB_V5_Ex is only used to validate the predicting model of our BRSNMP-based prediction method ("Comprehensive DDI prediction in the cold-start scenario" section).

Moreover, to verify our observation on the weakly balanced relationship among the drugs, we first make a statistics of triad types. Totally, the DDI network included in DB_V4 contains 50.96% PPP, 18.56% NNP, 7.11% NNN and 23.37% unbalanced PPN triads. Then, we investigate whether the subsampling of drugs influences the composition of the four triads. After removing a set of drugs (e.g. 1/20, 1/8, 1/4 and 1/2 drugs) randomly and the involving DDIs from DB_V4, we observe that the triad composition has no significant change. For instance, after we remove 1/8 drugs and their DDIs, the subnetwork of DDIs contains 51.18% PPP, 18.34% NNP, 7.16% NNN and 23.28% PPN triads. Last, we compare the DDI network with a randomized network, which is generated by randomly shuffling enhancive and degressive DDIs among the drugs. In such a randomized network, we observe a group of significant different triad compositions, which contain 55.6% balanced triads (including 33.1% PPP, 19.6% PNN, 2.9% NNN) and 44.4% unbalanced triads (PPN). The above pieces of evidence reveal that the real DDI network has an intrinsic property of weakly balanced relationship among drugs.

### Drug community partition

In this section, we investigate the communities generated by BRSNMF and compared them with those generated by Semi-NMF. Similar to the traditional clustering, *k*-means, either our BRSNFM or Semi-NMF require a parameter (*k*) to indicate the anticipated number of clusters in advance. In fact, clustering algorithms, no matter what they are, surely need a parameter to be specified. For example, centroid-based clustering algorithms (e.g. *k*-means, *k*-medoids, fuzzy *c*-means) need to specify the number of clusters (*k*); connectivity-based clustering algorithms (e.g. UPGMA) are able to provide a hierarchical clustering and still need a cutoff to determine the final clusters; distribution-based clustering algorithms (e.g. Gaussian mixture models) use a fixed number of distributions corresponding to the number of clusters; density-based clustering algorithms (e.g. DBSCAN and Mean-shift) define the clusters are areas of high density, which depends on a density criterion. Like *k*-means, once the number of communities, *k*, is given, BRSNMF splits samples into *k* non-overlapping groups. In the context of the comprehensive DDI network, BRSNMF partitions drugs into *k* communities.

#### Parameter tuning in community partition

Before performing the comparison, we check how the tuning parameters (*α*, *β*, *η*, and *σ*) in Formula 5 influence the clustering. Since *β* controls both *σ* and *η* (shown in Formula 5) simultaneously, we just tune *α* and *β* from 0.05, 0.25, 0.5, 1, and 5 respectively with fixing *σ* = 1 and *η* = 1.

First, we globally measure the influence by CBI (defined in Formula 10). By running the grid search of *α* and *β*, we obtain 25 values of CBI with each pair of *α* and *β* for a specific number of drug communities. Moreover, we measure the influence by two interaction ratio-derived items, including $SR_{{}}^{w} = R_{e}^{w} + R_{d}^{w}$ and $DR_{{}}^{w} = R_{e}^{w} - R_{d}^{w}$. The first one denotes how dense the community is, while the second one reflects whether enhancive DDIs or degressive DDIs are dominant. Again, we obtain 25 pairwise values of $SR_{{}}^{w}$ and $DR_{{}}^{w}$ for each drug community in the case of a specific number of drug communities. The influence of these parameters on drug partition is measured by their standard deviations. The smaller the standard deviation, the less sensitive the partition to the parameters.

In the case of k = 3, for example, BRSNMF splits samples into 3 non-overlapping drug communities, where both the first community and the third one are strongly balanced while the second one is weakly balanced. Overall, BRSNMF achieves CBI = 0.8958 ± 0.0080, which demonstrates that the balance across communities, on average, is less variable. On the other side, for the strongly balanced communities, their $SR_{{}}^{w}$ are 0.3318 ± 0.0151 and 0.1422 ± 0.0015. Meanwhile, their $DR_{{}}^{w}$ are 0.3057 ± 0.0181 and 0.1161 ± 0.0023. For the weakly balanced community, its $SR_{{}}^{w}$ and $DR_{{}}^{w}$ are 0.2938 ± 0.0225 and − 0.2307 ± 0.0282 respectively. These small standard deviations reflect that both the community dense and the dominant type of DDI in community changes trivially. Similar results are observed in other cases of *k* during the grid search of *α* and *β*. The experiments show that the generated communities in all the combinations of *α* and *β* are consistent.

To summarize, BRSNMF is robust to different values of parameters. Thus, for simplicity, we fixed all the tuning parameters with 1 (*α* = *β* = *η* = *σ* = 1).

#### Better drug community partitions achieved by BRSNMF

**Fig. 3:**
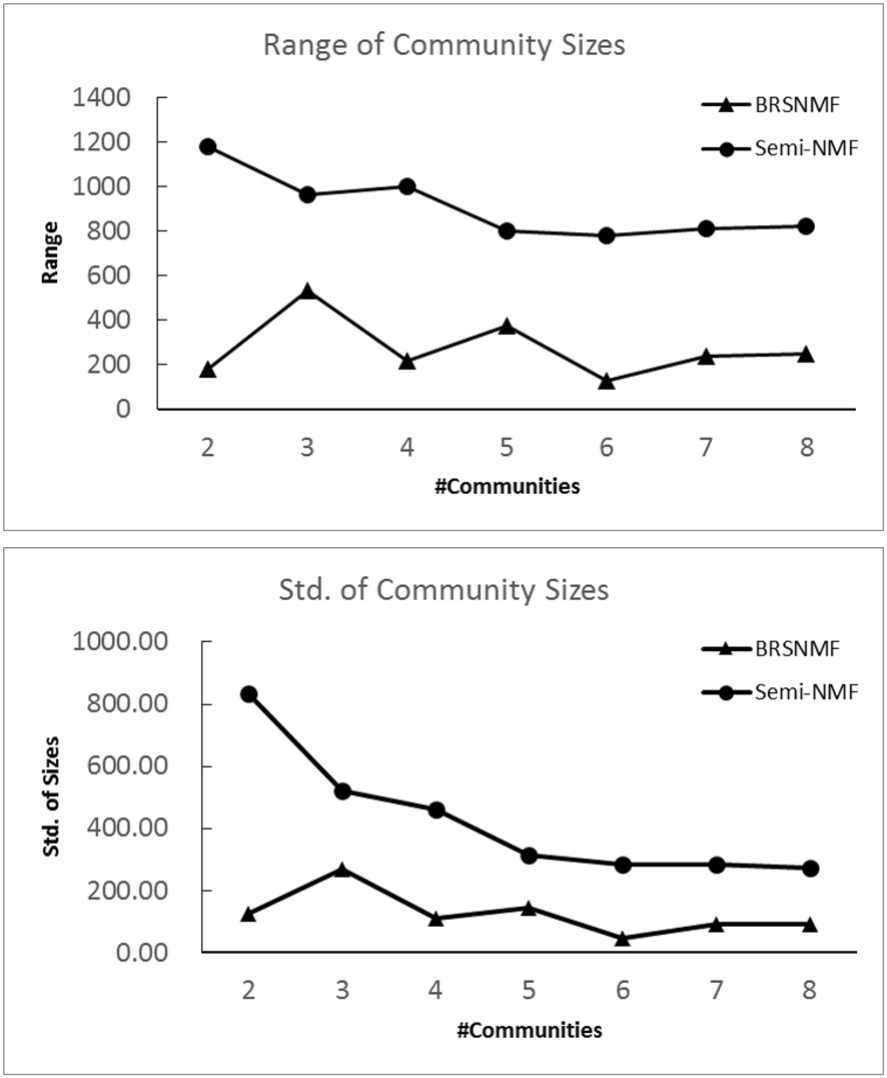
The statistics of community sizes

To demonstrate the superiority of BRSNMF, we run BRSNMF and Semi-NMF to partition the comprehensive DDI network into communities respectively. First, we investigate the community sizes (drug numbers in community) when given different community numbers, where *k* = 2, 3, 4, 5, 6, 7, and 8 respectively (Table 2). In terms of community size, both Range and Standard Derivative measure the community partition (clustering). The smaller the value, the better the partition. Compared with Semi-NMF, the results show that BRSNMF tends to generate the communities having both the smaller ranges and the smaller standard deviations significantly in terms of community size (Fig. 3 and Table 2). Specifically, all the communities generated by BRSNMF contain a substantial number of drugs, especially when *k* is large. For instance, in the case of *k* = 8, the smallest community generated by Semi-NMF contains only 7 drugs while that generated by BRSNMF contains 45 drugs. In short, BRSNMF is able to partition drugs into the communities, of which each contains enough drugs and the number of its drugs is less dispersed across all the communities.

**Table 2:** Summary of community sizes

k	Cluster Id	1	2	3	4	5	6	7	8
2	Semi-NMF	1372	190	–	–	–	–	–	–
	BRSNMF	870	692	–	–	–	–	–	–
3	Semi-NMF	1115	151	296	–	–	–	–	–
	BRSNMF	469	281	812	–	–	–	–	–
4	Semi-NMF	1072	160	260	70	–	–	–	–
	BRSNMF	340	262	480	480	–	–	–	–
5	Semi-NMF	849	147	313	46	207	–	–	–
	BRSNMF	318	252	397	111	484	–	–	–
6	Semi-NMF	804	129	323	105	178	23	–	–
	BRSNMF	274	247	305	270	178	288	–	–
7	Semi-NMF	823	115	312	108	170	25	9	–
	BRSNMF	258	232	361	122	134	299	156	–
8	Semi-NMF	827	110	304	110	147	31	7	26
	BRSNMF	277	224	45	84	171	293	189	279

**Fig. 4:**
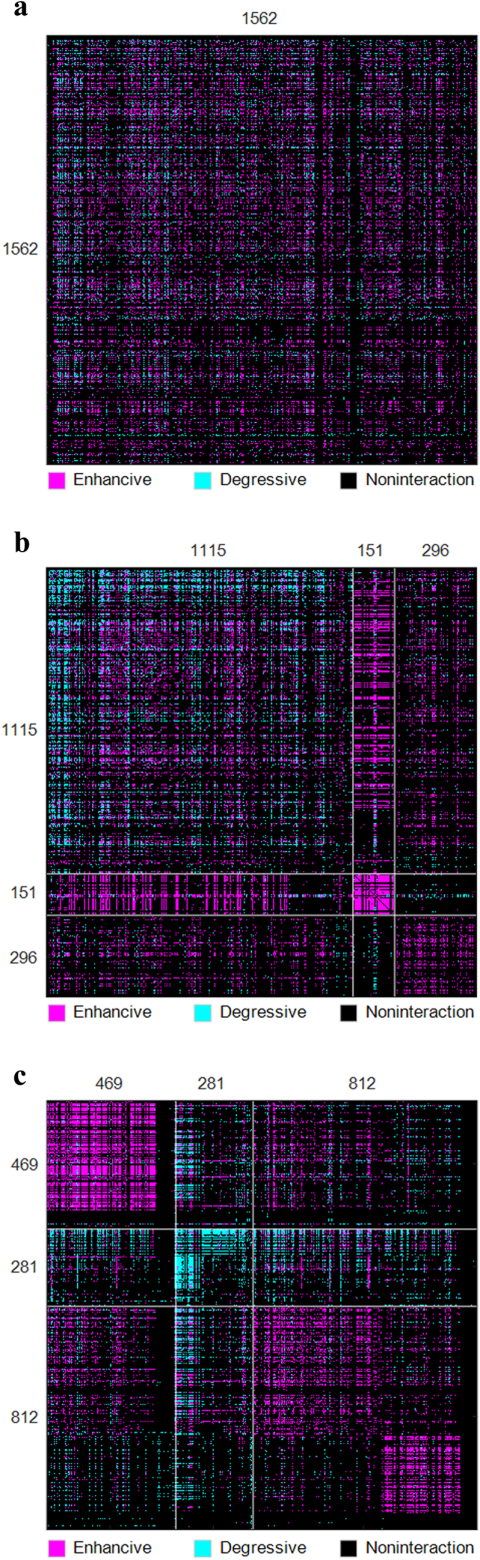
Community images of the DDI adjacent matrix. **a** Original DDI matrix, **b** rearranged DDI matrix upon three communities generated by Semi-NMF; **c** rearranged DDI matrix upon three communities generated by BRSNMF. Each pixel in the DDI matrix image represents a drug pair. Magenta pixels represent enhancive DDIs, cyan pixels represent degressive DDIs and black pixels are non-DDIs. The boundaries of communities are highlighted by white lines. The numbers of drugs in communities in all the images are exhibited as well. Semi-NMF generates the communities with larger variance of community sizes and cannot detect the weakly balanced community. In contrast, BRSNMF generates the communities which are less dispersed in terms of community size. More importantly, BRSNMF is able to detect a weakly balanced community

Moreover, we choose the case of three communities to take a deeper analysis, where Semi-NMF generated three communities containing 1115, 151 and 296 drugs respectively while BRSNMF achieved the communities containing 469, 281 and 812 drugs respectively. We measure the communities generated by two approaches in terms of the global metric, CBI, defined in Formula 10. Our BRSNMF achieves 89.58% while Semi-NMF achieves 82.54% in the case of 3 communities. We also measure them by two proposed local metrics, including community-within differences for each community and community-between differences for pairwise communities. The differences are grouped into matrices (Table 3), in which the diagonal entries list the values of Δ_*w*_ and the off-diagonal entries denote the values of Δ_*b*_. The results show that the average Δ_*w*_ of strongly balanced communities achieved by Semi-NMF and BRSNMF are 2.4437 and 2.7676 respectively and the average Δ_*b*_ are 1.0207 and 0.0952 respectively. According to our criteria about Δ_*w*_ and Δ_*b*_, BRSNMF is significantly superior to Semi-NMF (see also "Clustering by balance regularized semi-nonnegative matrix factorization" section ). In particular, except for two strongly balanced communities, BRSNMF is able to detect a weakly balanced community (its Δ_*w*_ < 0) whereas Semi-NMF cannot. Compared with the whole DDI network, such a weakly balanced community shows a special triad composition that contains 0.85% PPP, 27.44% NNP, 67.19% NNN, and only 4.52% unbalanced PPN triads. In addition, after reordering the DDI matrix according to the communities generated by Semi-NMF and BRSNMF respectively, we visualize these communities as two pseudo-color images, which provide an illustration consistent to Δ_*w*_ and Δ_*b*_ (Fig. 4b, c). Meanwhile, as a comparison, the original image of DDI matrix is also shown (Fig. 4a). In short, by capturing the intrinsic property of weakly balanced relationship among drugs, BRSNMF, compared with Semi-NMF, is able to generate a better drug partition, where drugs within a cluster (drug community) tend to exhibit the strongly or weakly balanced relationship while drugs belonging to two different clusters tend to show the unbalanced relationship.

**Table 3:** Comparison of community-within difference (Δ_*w*_) and community-between difference (Δ_b_**)**

Semi-NMF	C1	C2	C3
C1	*0.3588*	2.2338	1.4717
C2	2.2338	*3.5961*	− 0.6434
C3	1.4717	− 0.6434	*3.3763*

BRSNMF	C1	C2	C3
C1	*3.2832*	− 0.1795	0.7275
C2	− 0.1795	− *2.1615*	− 0.2624
C3	0.7275	− 0.2624	*2.2520*

Italic values indicate community-within difference while regular values indicate community-between difference

#### Pharmacological significance of balanced clusters

The generated clusters are valuable in clinics. Specifically, drugs attending in the multiple-drug treatment would cause pharmacological changes due to their interactions. The result of pharmacological changes can be deduced if the drugs come from the same balanced community (usually forming a balanced *l*-cycles), whereas it cannot be inferred if the drugs come from different communities. These pharmacological changes surely influence clinical medication, including dosage, medicine interval, therapeutic window, synergistic combination, and so on.

Furthermore, they are important to biology. The interaction between two drugs is always caused by their binding to common or functionally related proteins (DBP), which can be roughly grouped into target proteins and non-target proteins. Drug targets are the proteins, which are bound by drugs to result in a desirable therapeutic effect, while non-target proteins usually play varied roles, such as catalyzing chemical reactions involving a specific drug, shuttling drugs across cell membranes, or increasing the effectiveness of drug delivery to the target sites of pharmacological actions.

Their meaning, potential application and biological implication are depicted as follows.

1. Meaning of balanced clusters

Assume that the drugs attend in a three-drug treatment and all the pairwise interactions between them change their serum concentration (SC). Such as a pharmacological change is enough to elucidate the meaning of balanced cluster though DDIs trigger varied pharmacological changes (i.e. the change of bioavailability, distribution, …) in reality. In this context, an enhancive interaction reflects the increment of SC while a degressive interaction indicates the decrement. We show a theoretical analysis of how the pharmacological changes derived from the drugs in a balanced cluster can be deduced in terms of drug triads as follows.

In a strongly balanced cluster, the pharmacological change (i.e. dose) of any drug in a triad (ideally a PPP triad or an NNP triad) surely causes the consistent influence on the triad. Let *d*_*i*_, *d*_*j*_, and *d*_*k*_ be three drugs in a triad. When the triad is a PPP triad, the slightly increasing dose of any of these drugs would increase the SCs of all of them, because any of them boosts the others. When the triad is an NNP, where both the interaction *d*_*i*_–*d*_*j*_ and the interaction *d*_*i*_–*d*_*k*_ are degressive and the interaction *d*_*j*_–*d*_*k*_ is enhancive, the slightly increasing dose of *d*_*i*_ would decrease the SCs of *d*_*j*_ and *d*_*k*_ while the slightly decreasing dose of *d*_*j*_ or *d*_*k*_ would increase the SC of *d*_*i*_. Obviously, the changes on the NNP triad from two sides are consistent as well.

In a weakly balanced cluster, only the coinstantaneous changes of all drugs in a triad (ideally an NNN triad) can generate a consistent influence on the triad, or it generates an unpredictable influence. When the triad consisting of *d*_*i*_, *d*_*j*_ and *d*_*k*_ is an NNN triad, the slightly increasing dose of *d*_*i*_ would decrease the SCs of *d*_*j*_ and *d*_*k*_ however the degressive interaction between *d*_*j*_ and *d*_*k*_ could trigger an opposite influence on *d*_*j*_ or *d*_*k*_. Obviously, two conflicted influences from two sides would result in a final unpredictable influence. The only possible condition to generate consistent influence on the triad is to increase doses of *d*_*i*_, *d*_*j*_ and *d*_*k*_ with the right proportion to their original doses.

Remarkably, the dose change of drugs in an unbalanced triad (ideally a PPN triad) between two balanced clusters surely trigger unpredicted influences on the triad. The similar interpretation to that of NNN triads can be drawn, but there is no condition to generate a consistent influence on the triad.

Similarly, it is easy to make an extended interpretation of the pharmacological meaning in terms of balanced *l*-cycles, which follows the naïve multiplication rule that the product of all the signs of a cycle’s edges is positive.

2. Potential application of balanced clusters

The clusters can be directly applied with the consideration of drug intolerance. When multiple drugs in therapy are delivered throughout the body, any change triggered by their DDIs in the ADME (absorption, distribution, metabolism, and excretion) process would change their concentration in the blood.

In a strongly balanced cluster, for example, three drugs, Cyclosporine, Pravastatin, and Lovastatin forms a PPP triad, which increases their serum concentrations. Meanwhile, both of the first two have two degressive interactions with another drug, Efavirenz (an NNP triad). Since the pharmacological change of even one drug in the balanced triads definitely influences other drugs, a multiple-drug treatment (e.g. the prophylaxis of graft rejection) involving them should investigate whether their interactions break their individual therapeutic windows, which are the differences between their minimum effective concentration (MEC) and minimum toxic concentration (MTC) respectively. When the concentration of a drug within the blood is less than its MEC, the drug cannot give rise to the intended therapeutic effect. When its concentration is greater than its MTC, the drug will trigger an unintended adverse drug event.

In addition, the clusters can be used to find synergistic drugs. For example, the pairwise interactions among Fluvoxamine (an antidepressant), Pregabalin (an anticonvulsant drug used for epilepsy and generalized anxiety disorder) and Magnesium sulfate (an anticonvulsant for pre-eclampsia and eclampsia) in a strongly balanced cluster can boost their therapeutic efficacies (a PPP triad). Therefore, their combination can be a potential synergistic multiple-drug treatment.

In general, after integrating pharmacological knowledge of DDIs, these drug clusters can be applied to guide multiple-drug treatments, such as optimizing drug doses, alerting risks and discovering synergistic drugs.

3. Biological implication of balanced clusters

To understand the biological implication of the balanced clusters, we finally investigate both the drugs within clusters and those between clusters by exploiting DBPs, which play important roles in pharmacodynamic and pharmacokinetic processes of drugs. After counting the numbers of non-target proteins and target proteins binding to each drug respectively, we calculate the averages of those numbers in each com cluster. The average numbers (*a*_*n*_) of non-target proteins binding to a drug are 2.35, 5.16 and 3.17, while the average numbers (*a*_*t*_) of target proteins binding to a drug are 4.12, 2.87 and 2.64 in these three clusters respectively. The one-way analysis of variance across clusters on the two groups of numbers (with *p* value = 2.22e−16 and 1.68e−07 respectively) shows that the drugs in different drug communities bind to significantly different numbers of non-target and target proteins on average. In particular, the investigation reveals interesting aspects: (1) drugs in the only weakly balanced community (the second one) tends to bind more non-target proteins than target proteins; (2) drugs in the first strongly balanced community (containing 93.95% strongly balanced triads) tends to bind a fewer number of non-target proteins but more target proteins. This observation, largely revealing the underlying mechanism of forming DDI, inspires us to propose a predictive model of comprehensive DDIs in the cold-start scenario.

### Comprehensive DDI prediction in the cold-start scenario

Recall that we use drug-binding proteins (DBP) as features to perform DDI prediction (see also "BRSNMF-based approaches for predicting potential comprehensive DDIs of new drugs" section). Considering these proteins, we generate the protein-profile feature as follows. Each drug is represented as a $1 \times 1642$ binary vector, of which each element denotes whether or not a specific protein binds to it. Slightly different to community detection which focuses on balance structure DDI, DDI prediction emphasizes more on reconstruction error.

#### Parameter tuning in prediction

**Fig. 5:**
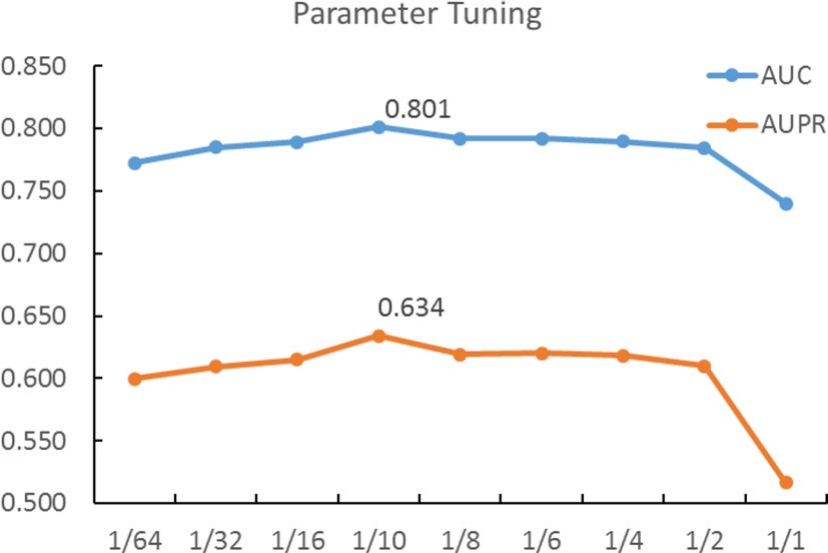
Tuning the best dimension of the latent space

Before running the subsequent comparison, we first investigate how the parameter *k* (the dimension of latent space) influences the prediction by tuning its value from the list {rank(A)* (1/64, 1/32, 1/16, 1/10, 1/8, 1/6, 1/4, 1/2, 1/1)}, where A is the training DDI matrix. The investigation on DB_V4 under 10-CV shows that the prediction is the best in the case of *k* = rank(A)/10 (Fig. 5). Meanwhile, this value also meets the need of low-rank matrix factorization. As a consequence, we use this value of *k* when performing the following cold-start prediction tasks, which require to infer the interactions between new drugs and approved drugs.

#### Cold-start DDI prediction boosted by DBP-based feature

To demonstrate the effectiveness of DBP, we compare the DBP feature with the popular PubChem fingerprint feature under both LOOCV and 10-CV. The comparison is performed on DB_V4. Here, we adopt PubChem fingerprints (V 1.3) to represent each drug as a $1 \times 881$ ordered binary vector, of which each element denotes whether a specific chemical substructure (fingerprint) is contained in the drug or not. These substructures involve hierarchic element counts, rings in a canonic extended smallest set of smallest rings, simple atom pairs, simple atom nearest neighbors, detailed atom neighborhoods, simple smarts patterns, and complex smarts patterns.

**Fig. 6:**
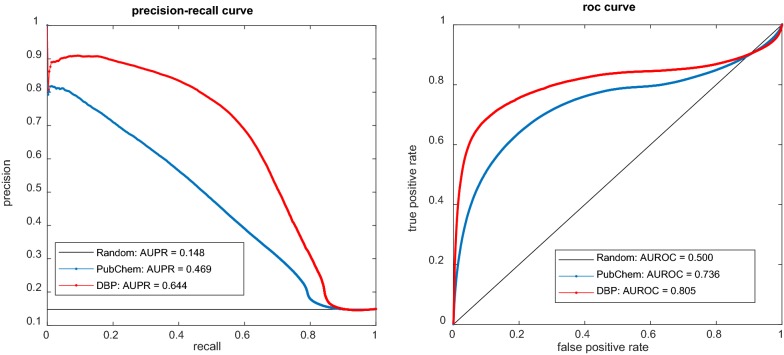
The comparison between DBP profile features and PubChem fingerprint features when predicting comprehensive interactions for newly given drugs

Both the ROC curve and the PR curve accounting for LOOCV are illustrated in Fig. 6. In addition, we make a comparison under 10-CV and measured the prediction by the average AUROC and the average AUPR in all the rounds of 10-CV. The prediction achieved by DBP achieves AUROC = 0.801 ± 0.019, AUPR = 0.634 ± 0.033 and MPR = 0.021 ± 0.017 while that achieved by PubChem fingerprints only achieves AUROC = 0.720 ± 0.018, AUPR = 0.455 ± 0.029 and MPR = 0.026 ± 0.018. The comparisons under both LOOCV and 10-CV show that DBP is greatly superior to the PubChem fingerprints.

The results demonstrate the superiority of DBP features. The underlying reason is that pharmacological DDIs are not direct or physical bindings, which are usually related to drug structures, but they are indirect interactions where DBPs play as the mediator. This nature of DDIs is quite different from that of drug–target interactions [35], which heavily rely on the direct binding between drug structures and protein pockets.

For example, the interactions between Ritonavir and Saquinavir are mediated by intestinal CYP3A4. In details, Ritonavir increases the bioavailability (the fraction of an administered dose of the drug that reaches the systemic circulation) of HIV protease inhibitors (e.g. Saquinavir), because it strongly inhibits the activity of intestinal CYP3A4 (an enzyme DBP), which acts as a metabolizer of these HIV protease inhibitors so as to influence their absorption [40]. Furthermore, we calculated the Pearson correlation coefficients (PCC) between Ritonavir and Saquinavir with DBP-based features (PCC = 0.5961) and fingerprint-based features (PCC = 0.3624) respectively. The greater the PCC value, the better the features. The result shows that DBP is better than PubChem fingerprint when capturing the association between Ritonavir and Saquinavir.

On the other side, we check whether the higher dimension of features achieves a better prediction. First, after analyzing them by PCA, we find that the effective dimension (426) of DBP is actually less than that (576) of PubChem Fingerprint, though the former’s original dimension is greater than that of the latter. In addition, using the concatenation of DBP-based features and PubChem fingerprint-based features, we perform an extra experiment under 10-CV. Compared with DBP-based features (AUROC = 0.801 ± 0.019 and AUPR = 0.634 ± 0.033), the result (AUROC = 0.804 ± 0.020 and AUPR = 0.636 ± 0.039) shows no significant improvement of DDI prediction. Obviously, compared with DBP, PubChem fingerprint doesn’t contain more information helpful to identify DDI. In short, the performance prediction doesn’t depend on the feature dimension but relies on the discriminant ability of feature, which reflects how well the feature can characterize DDI. Therefore, we believe that the proposed DBP-based feature is better than the popular fingerprint-based feature because the former is able to capture the nature of DDIs.

#### Accurate DDI prediction for new drugs by BRSNMF-based approach

To test the effectiveness of our BRSNMF-based approach in the real scenario of newly given drugs, we make a version-independent validation, which uses the drugs in DB_V4 as the training drugs and those in DB_V5_Ex as the independent testing drugs respectively. The drugs pairs in DB_V4 are taken as training drug pairs, while the testing drug pairs are the pairs between the drugs in DB_V4 and the drugs in DB_V5_Ex.

According to DrugBank, both the training pairs and the testing pairs have real labels, which indicate interactions. In other words, we know the labels of the interactions between the drugs in DB_V4 and the drugs in DB_V5_Ex. Thus, we use those labels in DB_V5_Ex (V5.0 updated on 2017-7-6) to measure the prediction. Totally, there are 78.8% balanced triads (including PPP, NNP and NNN) and 21.2% unbalance triads (PPN) within the DDIs between DB_V4 and DB_V5_Ex. Again, DBP is used as drug features when running both our BRSNMF-based approach and the state-of-the-art approach, DDINMF [21].

During measuring the predictions, we first sort the testing drug pairs according to their predicting scores (can be positive, negative or near zero) generated by the predictive approaches. Because the labels of enhancive DDIs, degressive DDIs and non-interactions are + 1, − 1, and 0 respectively, there are three expectations on predicting results. It is anticipated that (1) enhancive DDIs tend to have positive scores. The greater the predicting score, the higher the chance the drug pairs are enhancive DDIs; (2) degressive DDIs tend to have negative scores. The smaller the predicting score, the higher the chance the drug pairs are degressive DDIs; (3) non-interactions tend to have scores near to zero. The closer the value to zero, the higher the chance the drug pairs are non-interactive. In addition, the range of predicting scores also mainly depends on the value of parameter *k*. For example, the range of predicting scores generated by BRSNMF-based approach is [− 0.2873, 0.4691] in the case of *k* = 1 while that is [− 1.6770, 2.5097] in the case of *k* = rank(A)/2. The greater the value, the larger the range. Thus, it is inappropriate to set fixed cutoffs of scores to discriminant enhancive and degressive. We use the position in the sorted list of the testing drug pairs as the cutoff.

Then, top-*n* out of predicted DDIs are selected out and checked for enhancive DDIs. According to their real labels in DrugBank, the drug pairs with positive labels among the top-*n* candidates are counted. The accuracy of predicting enhancive DDIs is defined as the number of such drug pairs over *n*. Similarity, the number of drug pairs with negative labels among the bottom-*n* divided by *n* is just the accuracy of predicting degressive DDIs. In addition, since DrugBank updates itself every half year, considering some entries in DB_V5_Ex are updated, we further double check the prediction by the labels provided by the latest version of DrugBank (V5.1.1 updated on 2018-8-8).

Finally, the prediction performance is measured in the case of *n* = 5, 10, 20, 30, 40 and 50 respectively (see the detailed results in Additional file 1: VersionIndTest.xslx). The ratios of correctly predicted DDIs are reported to measure the performance of the test (Table 4). The results show our BRSNMF-based approach achieves 94% accuracy among top-50 enhancive candidates and 86% accuracy among bottom-50 degressive candidates respectively. Like the metrics used in LOOCV, we also report the values of both AUROC and AUPR as the overall performance in the novel prediction. The overall performance of prediction achieved by our BRSNMF is AUROC|AUPR = 0.645|0.346 whereas that achieved by DDINMF is AUROC|AUPR = 0.597|0.299. In summary, it is demonstrated that our BRSNMF is significantly superior to DDINMF in a real application.

**Table 4:** The ratios of correctly predicted DDIs in top-50 and bottom-50 candidates

Enhancive	Top 5 (%)	Top 10 (%)	20 (%)	30 (%)	40 (%)	50 (%)
DDINMF	40	60.0	80.0	83.3	85.0	88.0
BRSNMF	*100*	*80.0*	*90.0*	*90.0*	*92.5*	*94.0*

Degressive	Bottom 5 (%)	Bottom 10 (%)	20 (%)	30 (%)	40 (%)	50 (%)
DDINMF	100	80.0	75.0	80.0	80.0	82.0
BRSNMF	100	80.0	*80.0*	*83.3*	*85.0*	*86.0*

Italic values indicate the better results where BRSNMF outperforms DDINMF

Though both the prediction achieved by our BRSNMF in the top-50 and that in the bottom-50 are inspiring, it is noted that the overall performance of prediction can still be improved. For this reason, we investigate those incorrectly predicted DDIs. After checking them case by case, we dig out three causes of wrong predictions.

The first is named as false positive drug pairs, which are inaccurately labeled as DDIs in DrugBank Version 4 but correctly labeled as non-DDIs in DrugBank Version 5. For example, the older version of DrugBank records that Apraclonidine (a sympathomimetic used in glaucoma therapy) increases the atrioventricular blocking activities of Alprenolol and Bevantolol, whereas the newer version removes it.

The second one is, on the contrary, called as false negative drug pairs, which are wrongly labeled as non-DDIs in DrugBank Version 4 but are corrected as newly reported DDIs in DrugBank Version 5 (e.g. the pair of Valrubicin and Ciclosporin as well as the pair of Ergocalciferol and Calcitriol). As the newer version of DrugBank reports, Valrubicin (for treating bladder cancer) increases the nephrotoxic activities of Cyclosporine (a powerful immunosuppressant with a specific action on T-lymphocytes), while the combined therapy of Calcitriol and Ergocalciferol increases the risk or severity of adverse effects in the multiple-drug therapy.

The last one refers to missing DBPs. Some DBPs are not collected in DrugBank such that two interacting drugs (e.g. Ritonavir and Darunavir; Amiodarone and Sofosbuvir) have no common DBPs in the dataset. However, Ritonavir increases the bioavailability of Darunavir in fact, because it strongly inhibits the activity of intestinal CYP3A4 (a DBP), which acts as a metabolizer of Darunavir so as to influence its absorption in HIV therapy [40]. Similarly, the preferential binding of Amiodarone to Albumin (one of plasma proteins) forces Sofosbuvir to redistribute and bind to other unexpected proteins, such that an unexpected adverse effect (severe symptomatic bradycardia) occurs when Amiodarone joins into Sofosbuvir-containing HCV therapy [41].

Therefore, it is anticipated to improve the existing prediction by two ways in the coming future. One is to build a better dataset containing a fewer number of both false positive drug pairs and false negative drug pairs. Another is to recover missing DBPs or update DBPs for drugs.

## Conclusions

It is more useful to know whether or not a drug pair is an enhancive DDI or a degressive DDI than to know whether or not a drug pair is a DDI. Without considering the pharmacological changes caused by DDIs, most existing approaches only report a binary prediction. Furthermore, the occurrence of both enhancive and degressive DDIs is not random but follows a weakly balanced relationship. However, none of existing approaches investigates and leverages this intrinsic property, which is one of the most crucial steps to understand high-order DDIs (involving three or more drugs) when treating complex diseases [7].

In this work, after representing the comprehensive DDI network containing pharmacological changes as a signed network, we’ve leveraged its weakly balanced structure to design a novel algorithm of balance regularized semi-nonnegative matrix factorization (BRSNMF). First, the proposed algorithm has been directly applied to detect drug communities. The comparison with the traditional Semi-NMF shows that each of the drug communities achieved by BRSNMF contains substantial drugs and their sizes have less dispersion. More importantly, these communities exhibit the weakly balanced relationship among drugs as well as their pharmacokinetic and pharmacodynamic significance in terms of drug-binding proteins. This finding helps to understand how high-order DDIs work.

Secondly, focusing on the scenario of predicting DDIs for newly given drugs, BRSNMF has been used to design a predictive approach for comprehensive DDI prediction. The experiments under LOOCV and 10-CV show that our DBP features are much better than popular PubChem fingerprints because pharmacological DDIs are not structure-derived interactions between drugs, but indirect protein-mediated interactions. Moreover, the version-independent test demonstrates that our BRSNMF-based predictive approach achieved the inspiring prediction of comprehensive DDIs and outperforms the state-of-the-art approach DDINMF due to its explicit modeling of the weakly balanced relationship among drugs. This predictive approach helps screen DDIs with the change of pharmacological effects.

Finally, it is anticipated that the BRSNMF-based approach will be able to achieve better DDI prediction by the better dataset with a fewer of both false positive drug pairs and false negative drug pairs, as well as more drug features from other drug attributes, especially protein-related properties (e.g. protein-protein network, side effects, ATC) in the coming future.

## Additional file

**Additional file 1.** Predicted results of version-independent test.

## Abbreviations

DDI: drug–drug interaction
BRSNMF: balance regularized semi-nonnegative matrix factorization
DBP: drug-binding protein
NMF: nonnegative matrix factorization
Semi-NMF: semi-nonnegative matrix factorization
PLSR: partial least-squares regression
AUROC: the area under the receiver operating characteristic curve
AUPR: the area under precision–recall curve
CV: cross-validation
LOOCV: leave-one-out cross-validation
